# A Postoperative Complication of Takotsubo Syndrome in the Spinal Surgery: A Case Report

**DOI:** 10.7759/cureus.51034

**Published:** 2023-12-24

**Authors:** Sergio Ramírez-Aragón, Jorge Del Pino-Camposeco, Eliezer Villanueva-Castro, Obet Canela-Calderon, Juan Nicasio Arriada-Mendicoa, Juan Antonio Ponce-Gómez

**Affiliations:** 1 Department of Neurosurgery, Instituto Nacional de Neurología y Neurocirugía Manuel Velasco Suárez, Mexico City, MEX

**Keywords:** postsurgical complication, spinal surgery, spondylolisthesis, stress cardiomyopathy, takotsubo syndrome

## Abstract

We present the case of a 32-year-old woman with a diagnosis of lumbar root syndrome and spondylolisthesis, which is why she underwent surgery. Anterior discectomy and intersomatic box placement plus posterior fixation were performed with percutaneous transpedicular screws in L5-S1. At 24 hours of the procedure, the patient presents sustained hypotension, adding sudden and intense chest pain with neck irradiation, dyspnea, and diaphoresis, as well as electrocardiographic abnormalities and elevation of cardiac enzymes suggestive of an acute coronary syndrome, subsequently evidence of basal hypokinesis in the echocardiogram. After providing hemodynamic support and analgesic management, the symptoms were resolved, and the electrocardiogram (ECG) and cardiac enzymes were normalized, allowing an adequate postoperative evolution.

## Introduction

Takotsubo syndrome, also known as broken heart syndrome, apical hot air balloon syndrome, stress cardiomyopathy, or neurogenic stunned myocardium, was first described in Japan in 1990 [1]. It treats an acute clinical syndrome characterized by transient left ventricular systolic dysfunction (less than 21 days) in the absence of underlying coronary disease, often associated with an emotional or physical stressor episode, which can be identified in the previous days (one to five days). The presence of anomalies of segmental contraction that characteristically extend beyond the territory irrigated by an epicardial artery defines the syndrome [2]. Recently, the syndrome has been dichotomized into primary and secondary forms. The primary form is usually triggered by a psychological stress factor in the outpatient setting and often presents with chest pain and dyspnea. The secondary form is triggered by physical stress, often in the context of a critical illness or surgery, and presents with signs of heart failure, including pulmonary edema, arrhythmias, or cardiac arrest [3].

It has been noted in outpatient and inpatient scenarios, critical care units, and the perioperative environment [4]. However, very few case reports have been made describing the appearance of this syndrome as a complication of neurosurgery, specifically spinal [5].

The aim of this case report is to take into account that this complication can occur, although it is extremely rare. It is worth knowing how to identify it since it can have a fatal outcome.

## Case presentation

**Figure 1:**
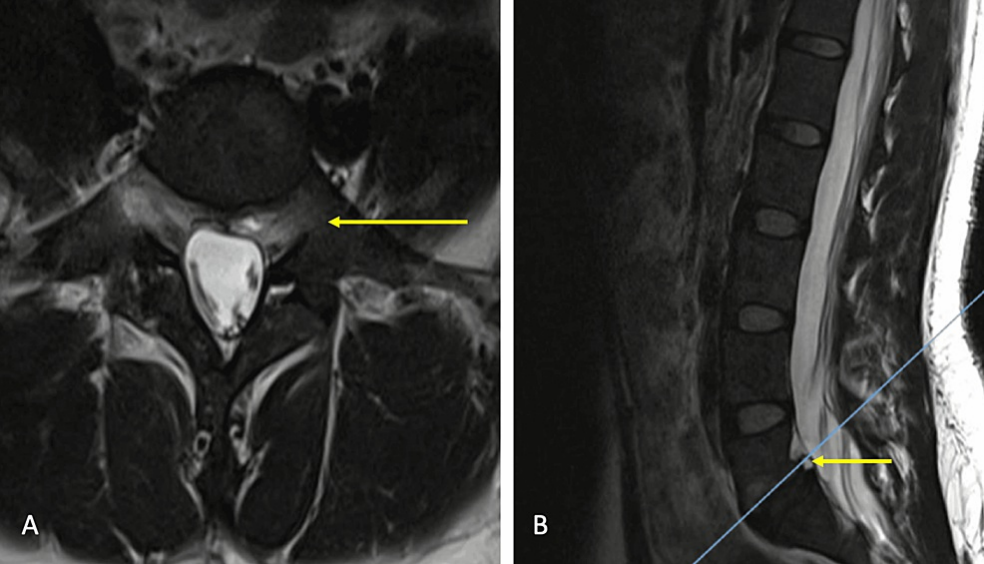
Preoperative imaging studies (A) Axial T2 MRI L5-S1 sequence without lumbar spinal stenosis, due to lytic spondylolisthesis (yellow arrow). (B) Sagittal T2 MRI L5-S1 sequence with grade 1 spondylolisthesis (yellow arrow). MRI: magnetic resonance imaging

This is a case report of a 32-year-old woman with a history of polycystic ovary syndrome since age 15 treated with metformin 850 mg every 12 hours with poor attachment, newly diagnosed dyslipidemia without treatment, and a complete vaccination scheme for SARS-CoV-2. She had a two-year history of back pain before her surgery, with an intensity of 7/10 on the visual analog scale (VAS), which evolved progressively with irradiation to the left gluteal region and then to the left pelvic limb in its entirety, with exacerbation to the effort; therefore, it is referred to our service where magnetic resonance imaging (MRI) is requested, in which spondylolisthesis grade I is observed (Figures 1A-1B), and likewise, an electrophysiological study with a report of damage in the left L5-S1 root. Surgical intervention is proposed.

We performed 360 fusion through an anterior left paramedian approach, retroperitoneal plus anterior discectomy, and intersomatic box placement plus posterior fixation with percutaneous transpedicular screws in L5-S1. The surgical procedure ended without incidents or complications, with a surgical time of 280 minutes and total bleeding of 200 mL. Upon discharge from the operating room, she presented the following vital signs: heart rate (HR) 59 beats per minute (bpm), blood pressure (BP) 125/74 mmHg, respiratory rate (RR) 16 respirations per minute (rpm), temperature (Temp) 36ºC, oxygen saturation (SaO_2_) 99%.

**Figure 2:**
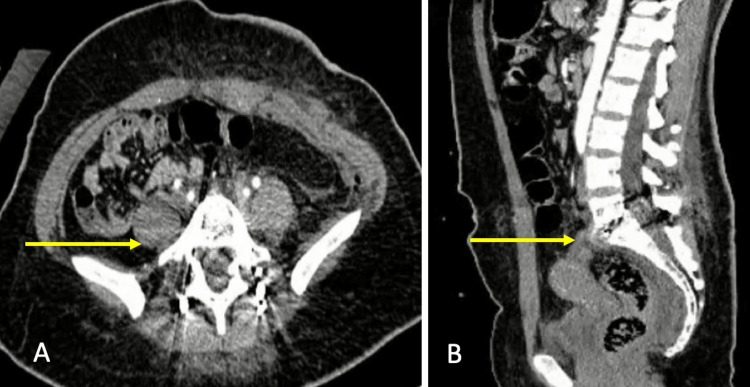
Postoperative imaging studies (A) CT axial section L5-S1 with proper placement of intervertebral cage and transpedicular screws (yellow arrow). (B) Sagittal CT section discarding free fluid and lesions of large vessels in the surgical site (yellow arrow). CT: computed tomography

At 24 hours, the patient presented the following vital signs: HR 90 bpm, BP 97/55 mmHg, RR 22 rpm, Temp 36.3ºC, SaO_2_ 92% with supplemental oxygen at 3 L/min, without hypoperfusion evidence, intravenous solutions were administered and angiotomography was performed, discarding free fluid and lesion of large vessels in the surgical site (Figures 2A-2B).

**Figure 3:**
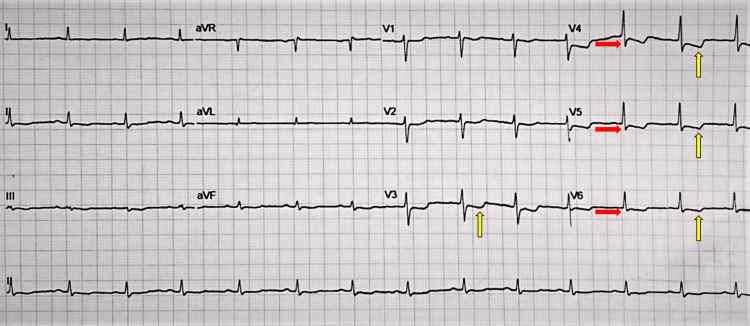
Electrocardiogram It shows ST segment depression >0.5mm in V4-V6 (red arrows) and inversion of the T wave in V3-V6 (yellow arrows).

Subsequently, she began with precordial pain of intensity 10/10 in VAS with irradiation to the right side and neck, adding dyspnea. The electrocardiogram (ECG) identified ST segment depression >0.5mm in V4-V6, T wave inversion in V3-V6, and the presence of isolated ventricular extrasystole (Figure 3); therefore, an urgent request for consultation to Cardiology was performed, nitrate therapy and analgesia with opiates were initiated due to suspected diagnosis of acute myocardial infarction without ST elevation (NSTEMI). A follow-up with a transthoracic echocardiogram (TTE) was performed, in which we observed adequate mobility of the left ventricle, parietal mobility index without systolic dysfunction, valvulopathies, and low probability of pulmonary hypertension. Hypotension showed no response to fluid therapy and presented a blood pressure of 60/40 mmHg, so it was decided to initiate vasopressor support with norepinephrine, as well as her admission to the Intensive Care Unit (ICU) and repeated TTE tracking, observing apical hypokinesia. Laboratories reported: creatine kinase fraction MB (CKMB) 21.9 ng/mL (reference range: 0.30-4.88), natriuretic peptide type B (NT-Pro BNP) 661.0 pg/mL (reference range: 5.0-134.1), and troponin T High Sensitivity 1234.0 (reference range: 3.0-14.0). 

## Discussion

Takotsubo syndrome was first described more than 30 years ago, and its perioperative appearance was recognized up to 10 years later. Reports have reported that 3-17% of cases have been associated with surgical procedures [5]. Many hypotheses have been generated around cause and pathogenesis; however, endogenous adrenergic augmentation is the most established theory and is intuitive given the strong association with sudden and unexpected stress and a major physical illness or trauma [6]. The prevalence of this syndrome in postmenopausal women is classically described due to the fact that relative estrogen deficiency results in certain endothelial dysfunction and a reduction in sympathetic tone [4,7]. Similarly, comorbidities associated with chronic coronary microvascular dysfunction, such as diabetes, asthma, and chronic obstructive pulmonary disease, appear to increase susceptibility to myocardial damage after a sudden increase in catecholamines, which may be secondary to an intense emotional stressor event (death of relatives, catastrophic events) or physical (acute respiratory failure, sepsis, surgery) [8]. It has been described that there is a higher prevalence in patients with neurological and psychiatric pathologies; for example, in the case of depression, it presents a secondary response to an exaggerated norepinephrine response to emotional stress [9]. The development of Takotsubo syndrome is a possibly serious complication of surgical procedures, especially in groups of patients at risk, particularly postmenopausal women; some factors that explain the appearance of this entity are a response to insufficiently controlled stress throughout the perioperative period, direct mechanical stimulation of the nervous system in selected surgeries [4], including a sudden surgical pain stimulus combined with insufficient analgesia; this can cause a sudden increase in catecholamines, which in turn, seems to cause microvascular spasms with subsequent dizziness and myocardial dysfunction; however, elevated levels of catecholamines in the blood are not always present [10].

Acute-onset chest pain, dyspnea, and changes in the ECG are clinically evident, and in extreme circumstances, patients may present with syncope, hemodynamic instability, severe heart failure, cardiogenic shock, or malignant arrhythmias [6,8].

ECG is abnormal in >95%; it initially appears an elevation of the ST segment, often deep and generalized T wave inversion, and significant QT interval prolongation, which usually develops 24 to 48 hours after the onset of symptoms, are quite specific for this pathology [11]. Takotsubo syndrome is considered an exclusion diagnosis; the criteria of the Mayo Clinic, first published in 2004 and modified in 2008, are the most recognized today and include the following points (Table 1):

**Table 1:** Mayo Clinic Takotsubo syndrome criteria Scantlebury et al. [12]. ECG: electrocardiogram

Takotsubo syndrome diagnosis criteria
1) Hypokinesis, akinesia, or transient dyskinesia of the middle segments of the left ventricle with or without apical involvement; regional wall motion anomalies extend beyond a single epicardial vascular distribution; a stressor trigger is often present, but not always.
2) Absence of obstructive coronary disease or angiographic evidence of acute plaque rupture.
3) New ECG abnormalities (ST-segment elevation and/or T-wave inversion) or moderate elevation of cardiac troponins.
4) Absence of pheochromocytoma or myocarditis.

A characteristic feature of this cardiomyopathy is the spontaneous resolution in hours or weeks; therefore, the treatment of the acute phase should be supportive, investigating factors or predisposing diseases, and if possible, making its correction; likewise, timely detection and appropriate treatment of possible complications, including arrhythmias, heart failure, pulmonary edema, cardiogenic shock, thromboembolism, and cardiac arrest, are essential [13]. Mortality was reported at 1.1%, with patients with physical stressors having a higher mortality rate than those triggered by emotional stressors; moreover, men are more likely to be precipitated by physical stressors [14], so mortality in men is higher.

## Conclusions

While recognition of Takotsubo syndrome as a postoperative complication has increased in recent years, many perioperative care providers are still unfamiliar with its presentation, this derived from a relatively low frequency, however, it should be considered and expected to appear in patients with and without risk factors compatible with clinical symptoms and/or alterations in ECG, cardiac enzymes, echocardiogram. Given the clinical and paraclinical similarities with acute coronary syndrome, it is essential to rule out the presence of the latter in order to establish timely medical treatment to prevent a fatal complication. Therefore, a multidisciplinary approach is indispensable in the diagnosis and management of this pathologic entity.

## References

[1] Boyd B, Solh T (2020). Takotsubo cardiomyopathy: review of broken heart syndrome. JAAPA.

[2] Aparisi Á, Uribarri A (2020). Takotsubo syndrome. Med Clin (Barc).

[3] Gibson LE, Klinker MR, Wood MJ (2020). Variants of Takotsubo syndrome in the perioperative period: a review of potential mechanisms and anaesthetic implications. Anaesth Crit Care Pain Med.

[4] Hessel EA 2nd (2016). Takotsubo cardiomyopathy and its relevance to anesthesiology: a narrative review. Can J Anaesth.

[5] Hessel EA 2nd (2021). Shining a light on perioperative Takotsubo syndrome. Can J Anaesth.

[6] Singh T, Khan H, Gamble DT, Scally C, Newby DE, Dawson D (2022). Takotsubo syndrome: pathophysiology, emerging concepts, and clinical implications. Circulation.

[7] Weaver J, Eubanks J (2013). Takotsubo cardiomyopathy following a L2-L5 laminectomy and fusion in situ with bone morphogenic protein. Case Rep Orthop.

[8] Del Buono MG, Potere N, Chiabrando JG, Bressi E, Abbate A (2019). Takotsubo syndrome: diagnostic work-up and clues into differential diagnosis. Curr Opin Cardiol.

[9] Díaz-Navarro R (2021). Takotsubo syndrome: the broken-heart syndrome. Br J Cardiol.

[10] Hammer N, Kühne C, Meixensberger J, Hänsel B, Winkler D (2015). Takotsubo cardiomyopathy - an unexpected complication in spine surgery. Int J Surg Case Rep.

[11] Kurisu S, Inoue I, Kawagoe T (2004). Time course of electrocardiographic changes in patients with tako-tsubo syndrome: comparison with acute myocardial infarction with minimal enzymatic release. Circ J.

[12] Scantlebury DC, Prasad A (2014). Diagnosis of Takotsubo cardiomyopathy. Circ J.

[13] Y-Hassan S, Tornvall P (2018). Epidemiology, pathogenesis, and management of Takotsubo syndrome. Clin Auton Res.

[14] Zhang Z, Kong H, Zhang SY, Guan TT (2021). Takotsubo syndrome triggered by change in position in a patient with thoracic vertebral fracture: a case report. Medicine (Baltimore).

